# Induction of an acute vitamin A-deficient state following total body irradiation impairs anti-tumor immunity by altering the homeostasis of pre-cDC derived dendritic cells

**DOI:** 10.1186/2051-1426-1-S1-P121

**Published:** 2013-11-07

**Authors:** Christopher A Klebanoff, Sean Spencer, Rahu Roychoudhuri, Yun Ji, Madhu Sukumar, Pawel Muranski, Joseph L Napoli, Luca Gattinoni, Yasmine Belkaid, Nicholas P Restifo

**Affiliations:** 1 CCR/NCI, Bethesda, MD, USA; 2 NIAID/NIH, Bethesda, MD, USA; 3 Program in Metabolic Biology, Nutritional Science and Toxicology, University of California, Berkley, CA, USA

## 

Host conditioning with total body irradiation (TBI) can potently augment adoptive T cell (ACT) immunotherapies by removal of sinks for homeostatic cytokines such as IL-7 and IL-15, depletion of CD4^+^ Tregs, and liberation of endogenous toll like-receptor agonists. Clinically, TBI-conditioning prior to ACT has been associated with durable complete responses in nearly 40% of patients with metastatic melanoma. However, TBI-conditioning also leads to dose-dependent mucosal injury and significant weight loss in the majority of treated patients. To explore the potential immunologic impact of this acute metabolic and nutritional perturbation, we profiled circulating micro-nutrients in the serum of TBI-conditioned patients and tumor-bearing mice receiving ACT. Unexpectedly, we found that TBI induced an acute vitamin A deficient (VAD) state. Immunologically, this state was associated with a selective loss in the splenic CD11b^+^CD8α^-^Esam^high^ (CD11b^+^) DC subset, the predominant DC subset in the spleen under steady state conditions which is specialized for class II antigen processing and presentation. By contrast, the CD11b^-^CD8α^+^ (CD8α^+^) DC subset, which is specialized for Ag cross-presentation to CD8^+^ T cells, was unaffected. Provision of exogenous retinoic acid (RA), the activated metabolite of vitamin A, restored the CD11b^+^ DC population following TBI. Further, evaluation of mice maintained on a VAD diet also demonstrated a CD11b^+^ DC defect that could be rescued by RA add-back, confirming that vitamin A is required in the physiologic maintenance of the CD11b^+^ subset. Mechanistically, VAD did not impair the formation of pre-DCs, the immediate hematopoetic progenitor of both the CD11b^+^ and CD8α^+^ DC subsets. Rather, in fate-tracking experiments, transfer of pre-DC into RA-supplemented hosts resulted in near complete conversion into the CD11b^+^ subset whereas transfer into VAD hosts caused diversion to the CD8α^+^lineage. Functionally, TBI-induced vitamin A deficiency resulted in impaired antitumor immunity mediated by ACT of CD4^+^ but not CD8^+^ T cells which could be rescued by supplemental RA. These findings establish a critical role for vitamin A in regulating the homeostasis of pre-DC derived DC subsets. Moreover, these data demonstrate how the nutritional and metabolic status of the tumor-hearing host can have a profound influence on therapeutic outcomes to vaccine and adoptive T cell immunotherapies.

